# Arp2/3 complex controls T cell homeostasis by maintaining surface TCR levels via regulating TCR^+^ endosome trafficking

**DOI:** 10.1038/s41598-017-08357-4

**Published:** 2017-08-21

**Authors:** Ye Zhang, Hao Shen, Haifeng Liu, Haiyun Feng, Yan Liu, Xiaoyan Zhu, Xiaolong Liu

**Affiliations:** 1 State Key Laboratory of Cell Biology, CAS Center for Excellence in Molecular Cell Science, Institute of Biochemistry and Cell Biology, Shanghai Institutes for Biological Sciences, Chinese Academy of Sciences, Shanghai, 200031 China; 2 University of Chinese Academy of Sciences, 320 Yue-Yang Road, Shanghai, 200031 China; 3 School of Life Science and Technology, ShanghaiTech University, Shanghai, 200031 China

## Abstract

T cell receptor (TCR) signaling is important for T cell homeostasis and function. However, how surface TCR levels are regulated and its biological significance on T cells remains largely unknown. Here, we show that the T cell-specific deletion of Arpc2, a component of Arp2/3 complex, results in compromised peripheral T cell homeostasis. Arp2/3 complex-nucleated actin filaments are essential for maintaining surface TCR levels by regulating TCR^+^ endosome trafficking in resting state and controlling polarization of TCR^+^ endosomes during immune synapse formation in T cells. Additionally, *Arpc2-*TKO T cells are unable to form immune synapse. Interestingly, defected T cell homeostasis is caused by reduced surface TCR levels but not impaired immune synapse formation. Collectively, our findings suggest that Arp2/3 complex-nucleated actin filaments are required for maintaining surface TCR levels via regulating TCR^+^ endosome trafficking which is essential for T cell homeostasis.

## Introduction

Peripheral T cells are maintained at a constant cell number so that they can efficiently recognize foreign antigens and protect the host from pathogen invasion. T cell homeostasis requires the combined regulation of T cell survival and proliferation, which are mediated by complex homeostatic signals^1–3^. It is widely accepted that there are two such homeostatic signals. One is an IL-7-induced signal that is essential for the survival and homeostatic proliferation of T cells. This signal activates the JAK/Stat5 pathway and increases the expression of the anti-apoptotic molecule B-cell leukemia/lymphoma 2 (Bcl-2)^4, 5^. The other is a self-peptide-recognition-induced basal TCR signal, which is also required for both T cell survival and homeostatic proliferation^6^. Previous studies have shown that the adoptive transfer of naïve T cells into major histocompatiblity complex (MHC) deficient mice resulted in shortened lifespans in both CD4^+^ and CD8^+^ naïve T cells, indicating that interactions between TCR and the self-peptide-MHC complex is necessary for T cell homeostasis^7, 8^.

The regulation of TCR expression level is essential for TCR signaling^9^. Synthesis, endocytosis, recycling and degradation are important processes that modulate and maintain TCR level in T cells^10^. In resting state, surface TCR maintenance relies on the constant transport of TCR between the intracellular pool and the plasma membrane^10^. TCR stimulation induces the downregulation of surface TCR levels by elevating its rate of endocytosis and the internalized TCR can then be recycled back to the plasma membrane^11^. By forming immune synapse (IS), which is a junctional structure located at the contact regions between T cells and antigen-presenting cells (APCs), T cells can rapidly accumulate TCR at the interface^12^. The contact region on T cells contains receptors, signaling proteins and cytoskeleton, forming a central supramolecular activation cluster (cSMAC) surrounded by 2 rings peripheral supramolecular activation cluster (pSMAC) and distal supramolecular activation cluster (dSMAC)^12–14^. During the initiation and maintenance of immune synapse, TCR recruitment to the contact area is quite important^14^. Previous studies have demonstrated that this recruitment requires surface TCRs to move toward the center of the immune synapse^14, 15^. Recently, some studies have investigated the mechanisms by which recycled TCR^+^ endosomes are delivered from the intracellular pool to immune synapse, a process that also plays a critical role in TCR recruitment^16^. In addition, this delivery ensures a steady supply of TCRs to the plasma membrane to sustain T-cell activation^17^. Finetti *et al*. showed that IFT20, a subunit of the intraflagellar transport (IFT) system, is required for the polarized recycling of TCR to immune synapse^18^. Recently, experiments that induced the loss of a series of Rab GTPase subunits such as Rab8, Rab11 and Rab29, also resulted in impaired TCR polarization during immune synapse formation^19–21^. Although TCR is required for homeostatic signals, the mechanisms that maintain surface TCR levels and its biological effects on T cell homeostasis remain largely unknown.

Actin-related Protein (ARP2/3) is a complex that is highly conserved in all eukaryotes that can nucleate branched actin filaments (F-actin) and generate actin networks^22, 23^. The Arp2/3 complex is a seven-member complex that includes Arp2 and Arp3, which are structurally similar to actin monomers and contact with actin. The other 5 subunits of the Arp2/3 complex are Arpc1, Arpc2, Arpc3, Arpc4 and Arpc5^23^. Arpc2 and Arpc4 form the core of the Arp2/3 complex and act to stabilize the complex^24^. By using specific small-molecule inhibitors or loss-of-function genetic manipulations, the Arp2/3 complex has been found to play an essential role in many biological processes^25^. For example, the Arp2/3 complex provides the protrusive force that is required to generate lamellipodia at the leading edge of cells^26^. Besides, the Arp2/3 complex regulates cell-cell junctions to promote the formation of epidermal barriers^27^. In Drosophila, the Arp2/3 complex helps to maintain Notch signaling by regulating the vesicles of the Notch ligand^28^. In the brains, the Arp2/3 complex plays an essential role in shaping and maintaining dendritic spines^29^. In T cells, TCR signal transduction can activate Nucleation Promoting Factors (NPF) to trigger the Arp2/3 complex-mediated actin polymerization^30^. Although the biochemistry and structure of the Arp2/3 complex has been extensively studied, the function of this complex in T cells has not been well characterized.

To investigate the specific role of the Arp2/3 complex in T cells, we generated *Arpc2*-TKO mice by specifically deleting of Arpc2 in T cells, which disrupted the integrity of the complex. We found that loss of Arpc2 caused a dramatic decrease in peripheral T cell numbers and compromised T cell homeostasis. In the absence of Arpc2, peripheral T cells exhibited impaired surface TCR maintenance and immune synapse formation. The impaired surface TCR maintenance was caused by defective TCR^+^ endosome trafficking, which is promoted by Arp2/3 complex-nucleated actin filaments and such compromised actin filaments were also accounted for defective polarization of TCR^+^ endosome during immune synapse formation. Moreover, our data demonstrate that the defective homeostasis observed in *Arpc2*-TKO T cells is caused by a reduction in surface TCR levels but not impaired immune synapse formation. Taken together, our research supports an essential role for the Arp2/3 complex in surface TCR maintenance and such TCR levels are critical for T cell homeostasis.

## Results

### Deficiency in Arpc2 leads to reduced numbers of peripheral T cells

**Figure 1:**
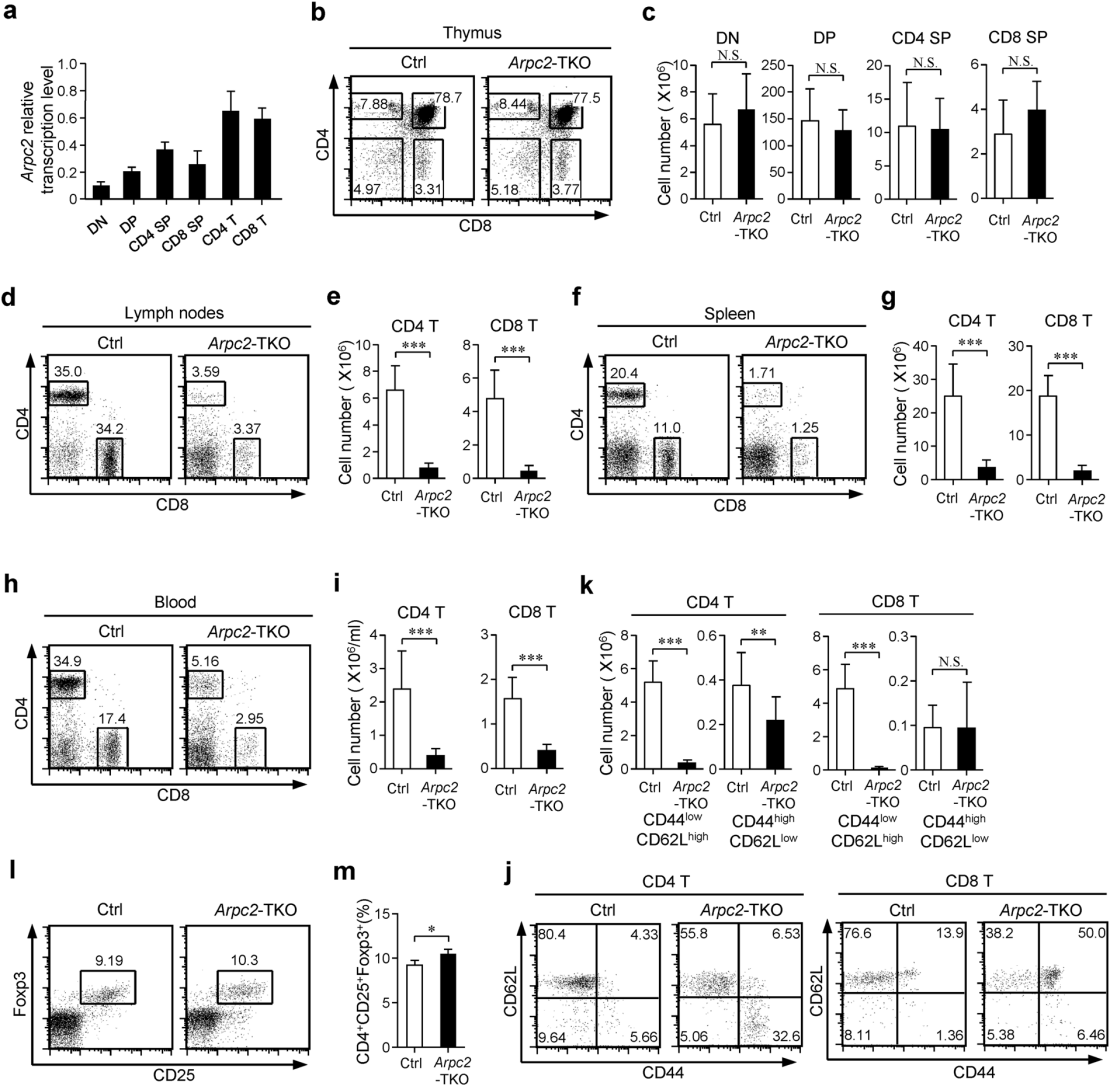
Targeted disruption of Arpc2 leads to a dramatic decrease in peripheral T cell numbers but does not influence thymocytes development. (**a**) Relative transcription level of *Arpc2* in DN, DP, CD4 SP, CD8 SP (n = 5), CD4 T and CD8 T cells (n = 6) in Ctrl mice. (**b**) Thymocytes obtained from Ctrl and *Arpc2*-TKO mice were analyzed for CD4 and CD8 expression using Flow cytometry. (**c**) Total number of CD4^−^ CD8^−^ double-negative (DN), CD4^+^ CD8^+^ double-positive (DP), CD4^+^ single-positive (CD4 SP) and CD8^+^ single-positive (CD8 SP) thymocytes (n = 8). (**d–i**) Flow cytometry analysis of CD4 and CD8 expression in (**d**) lymph nodes, (**f**) spleen and (**f**) blood obtained from Ctrl and *Arpc2*-TKO mice. Total number of CD4^+^ and CD8^+^ T cells in (**e**) lymph nodes (n = 11), (**g**) spleen (n = 11) and (**i**) blood (n = 7) are illustrated. (**i**) The expression of CD44 and CD62L on lymphocytes. (**k**) The total number of CD4^+^ and CD8^+^ naïve (CD44^low^CD62L^high^) and effector/memory (CD44^high^CD62L^low^) T cells in the lymph nodes was quantified in Ctrl and *Arpc2*-TKO mice. (n = 11). (**l**) Flow cytometry analysis of CD25 and Foxp3 in gated CD4^+^ T cells obtained from lymph nodes of Ctrl and *Arpc2*-TKO mice. (m) The percentage statistics for Treg cells (CD4^+^CD25^+^Foxp3^+^). (n = 3). The data are means ± S.D., for all panels: *P < 0.05; **P < 0.01; ***P < 0.001 by Student’s *t*-test, N.S.: no significance. All results are representative from at least three independent experiments.

Arpc2 and Arpc4 were demonstrated to form the core of the Arp2/3 complex. Point mutations in Arpc2 disrupted the activity of Arp2/3 complex-promoted branched actin nucleation^23, 31^. We examined the expression pattern of Arpc2 in different subsets of T cells. Intriguingly, peripheral T cells exhibited higher Arpc2 transcription and protein level than were observed in thymocytes (Fig. 1a and Supplementary Fig. S1a). Thus, we speculated that Arpc2 might play an essential role in peripheral T cells. To examine this hypothesis, we bred conditional *Arpc2* ^L/L^ mice^32^ with *Cd4*-*cre* mice. This breeding resulted in the specific deletion of Arpc2 from double-positive (DP) stage of T cells in the thymus in *CD4-cre* ^+^ *Arpc2* ^L/L^ mice (designated *Arpc2*-TKO mice) (Supplementary Fig. S1b-d). Compared with their wild-type control littermates (designated Ctrl mice, *Arpc2* ^L/L^ or *Arpc2* ^L/+^), the expression of Arp2/3 complex component Arp3 was also decreased in *Arpc2*-TKO T cells, indicating that the integrity of the Arp2/3 complex was compromised (Supplementary Fig. S1e). The frequency and absolute number of various T cell subsets in the thymus of *Arpc2*-TKO mice were not significantly different from those in Ctrl mice (Fig. 1b,c). The expression of the TCR signaling strength markers CD5^1, 6^ were similar between Ctrl and *Arpc2*-TKO DP and SP thymocytes (Supplementary Fig. S1f). The expression of TCRβ was equal between Ctrl and *Arpc2*-TKO DP thymocytes and slightly lower in *Arpc2*-TKO SP thymocytes (Supplementary Fig. S1g). However, the frequencies and absolute numbers of peripheral CD4^+^ and CD8^+^ T cells were lower in the lymph nodes, spleen and blood in *Arpc2*-TKO mice than in Ctrl mice (Fig. 1d-i). The cellularity of lymph nodes, spleen was also decreased (Supplementary Fig. S2a). There was no difference in the absolute numbers of B cells in the lymph nodes and spleen (Supplementary Fig. S2b-d). Moreover, we found that the loss of Arpc2 led to a significant decrease in naïve T cells population compared with Ctrl mice (Fig. 1j,k). The decreased number of peripheral T cells in *Arpc2*-TKO mice may have resulted from an increasement in Tregs. However, the proportion of Tregs in the *Arpc2*-TKO mice was similar to that in Ctrl mice (Fig. 1l,m). Together, these data suggest that the loss of Arpc2 leads to a significant decrease in peripheral T cell numbers but does not influence thymocyte development.

### T cell homeostasis is impaired in the absence of Arpc2

**Figure 2:**
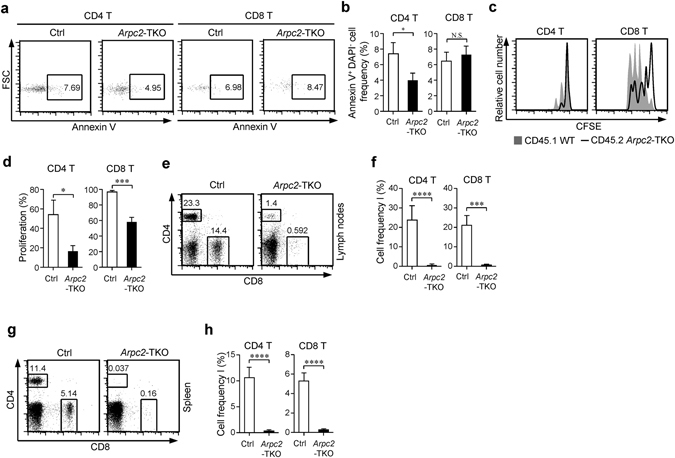
The loss of Arpc2 leads to impaired T cell homeostasis. (**a**) Survival was assessed in naïve T cells using flow cytometry analysis with Annexin V and DAPI staining. (**b**) The percentage statistics for Annexin V of naïve T cells obtained from Ctrl and *Arpc2*-TKO mice are illustrated. (n = 3). (**c**) Naïve CD4^+^ or CD8^+^ T cells isolated from CD45.2^+^ *Arpc2*-TKO mixed equally with CD45.1^+^ congenic Ctrl mice were labeled with carboxyfluorescein succinimidyl ester (CFSE) and co-transferred into *Rag1* ^−/−^ mice. After 7 days the mice were sacrificed, and the lymphocytes were analyzed. (**d**) The percentage statistics for CFSE dilution proliferated CD4^+^ and CD8^+^ T cells was analyzed (n = 3). (**e-h**) A mixture of equal number of CD45.1^+^ Ctrl and CD45.2^+^ *Arpc2*-TKO bone marrow cells was transferred into *Rag1* ^−/−^ mice. Flow cytometry analysis of CD4 and CD8 expression of T cells in (**e**) lymph nodes and (**g**) spleen. The percentage statistics for CD4^+^ and CD8^+^ T cells in (**f**) lymph nodes (n = 4) and (**h**) spleen (n = 6). The data are means ± S.D., for all panels: *P < 0.01; ***P < 0.001; ****P < 0.0001 by Student’s *t*-test, N.S.: no significance. All results are representative from at least three independent experiments.

Given that survival and proliferation are both critical for maintaining peripheral T cell number^1^, we analyzed survival and proliferation of T cells in the absence of Arpc2. Compared with Ctrl mice, the apoptosis of *Arpc2*-TKO CD4^+^ naïve T cells was lower and the apoptosis of *Arpc2*-TKO CD8^+^ naïve T cells was similar (Fig. 2a,b). Next, we examined the homeostatic proliferation potential of *Arpc2*-TKO naïve T cells using adoptive transfer assay^33^. As expected, the recovery of *Arpc2*-TKO naïve T cells was much slower than Ctrl mice (Fig. 2c,d). Additionally, by generating bone-marrow chimeras, we found that Arpc2 affected peripheral T cell homeostasis in a cell-intrinsic manner (Fig. 2e-h). Collectively, these results indicate that Arpc2 is essential for peripheral T cell homeostasis.

### TCR signaling is attenuated after Arp2/3 complex ablation

**Figure 3:**
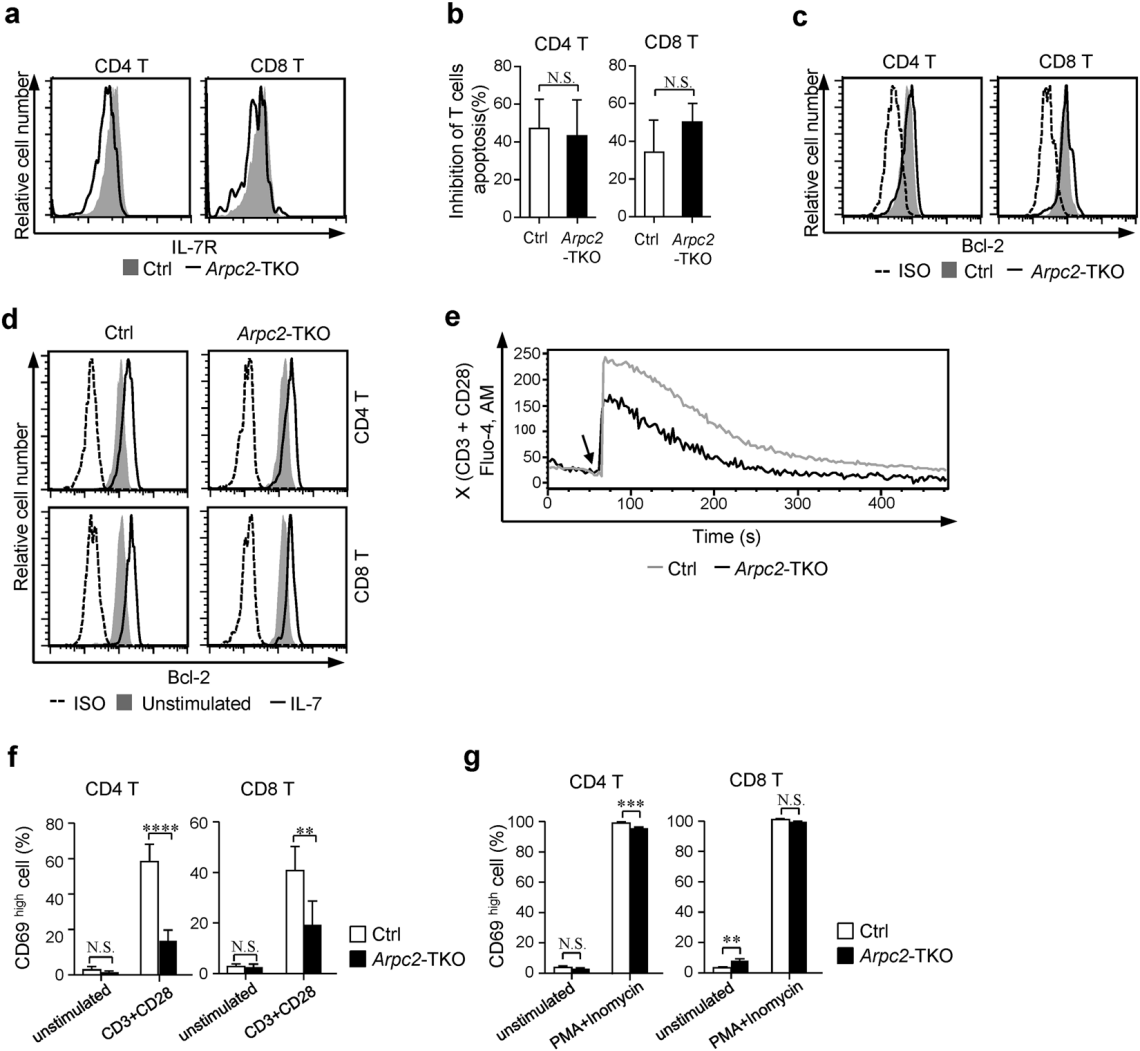
Disrupting the Arp2/3 complex in T cells resulted in attenuated TCR signaling. (**a**) Flow cytometry analysis of surface IL-7R expression in lymphocytes. Representative pictures are shown. (**b**) Freshly isolated lymphocytes were cultured with 0.25 ng/ml IL-7 for 36 h, then stained with anti-CD4 and anti-CD8 antibodies followed by staining with Annexin V. They were analyzed using flow cytometry. (n = 5). (**c**) Flow cytometry analysis of Bcl-2 expression in T cells. Mouse IgG1, κ isotype control were used. (**d**) Freshly isolated T cells were cultured with 5 ng/ml IL-7 for 24 h and the expression level of Bcl-2 was analyzed using Flow cytometry. (**e**) Naïve CD4 T cells obtained from Ctrl and *Arpc2*-TKO mice were loaded with Fluo-4, AM and then stained with anti-CD4-APC, biotinylated anti-CD3and anti-CD28. After they were cross-linked with streptavidin, the gated CD4-positive naïve T cells were analyzed for Ca^2+^ mobilization. Arrows indicate the time point at which the streptavidin was added. (**f**) Flow cytometry analysis of CD69 expression in T cells that were unstimulated or stimulated with anti-CD3 and anti-CD28 activator beads at a 2:1 (cell: bead) ratio for 24 h (n = 3). (**g**) Flow cytometry analysis of CD69 expression in T cells that were unstimulated or stimulated with PMA/Ionomycin for 4 h (n = 3). The data are means ± S.D., for all panels: *P < 0.05; **P < 0.01; ***P < 0.001; ****P < 0.0001 by Student’s *t*-test, N.S.: no significance. All results are representative from at least three independent experiments.

Because IL-7-induced signals are required for T cell homeostasis^4, 5^, we examined surface IL-7 receptor (IL-7R) level. Although the surface expression of IL-7R was slightly decreased in *Arpc2*-TKO naïve T cells (Fig. 3a), these cells responded equally to IL-7-induced inhibition of T cell apoptosis compared with Ctrl mice (Fig. 3b). The expression of Bcl-2 was equal between Ctrl and *Arpc2*-TKO naïve T cells (Fig. 3c), and the upregulation of Bcl-2 following IL-7 stimulation was normal in *Arpc2*-TKO naïve T cells (Fig. 3d). As previous work demonstrated that TCR recognition of self-ligand-MHC complex was essential for T cell homeostasis^34^, we next examined TCR signaling. Notably, the level of TCR-induced calcium flux was reduced in *Arpc2*-TKO naïve T cells compared with Ctrl mice by anti-CD3 and anti-CD28 crosslink (Fig. 3e). The upregulation of CD69 expression of *Arpc2*-TKO naïve T cells was diminished following TCR stimulation (Fig. 3f and Supplementary Fig. S3a) but similar when cells were stimulated with phorbol myristate acetate (PMA)/ionomycin (Fig. 3g), which bypass receptor-proximal signaling^35^. Presumably, the defective T cell homeostasis that was observed in cells lacking Arpc2 might be caused mainly by a reduction in TCR-proximal signals.

### Ablation of the Arp2/3 complex results in impaired surface TCR maintenance and immune synapse formation in peripheral T cells

**Figure 4:**
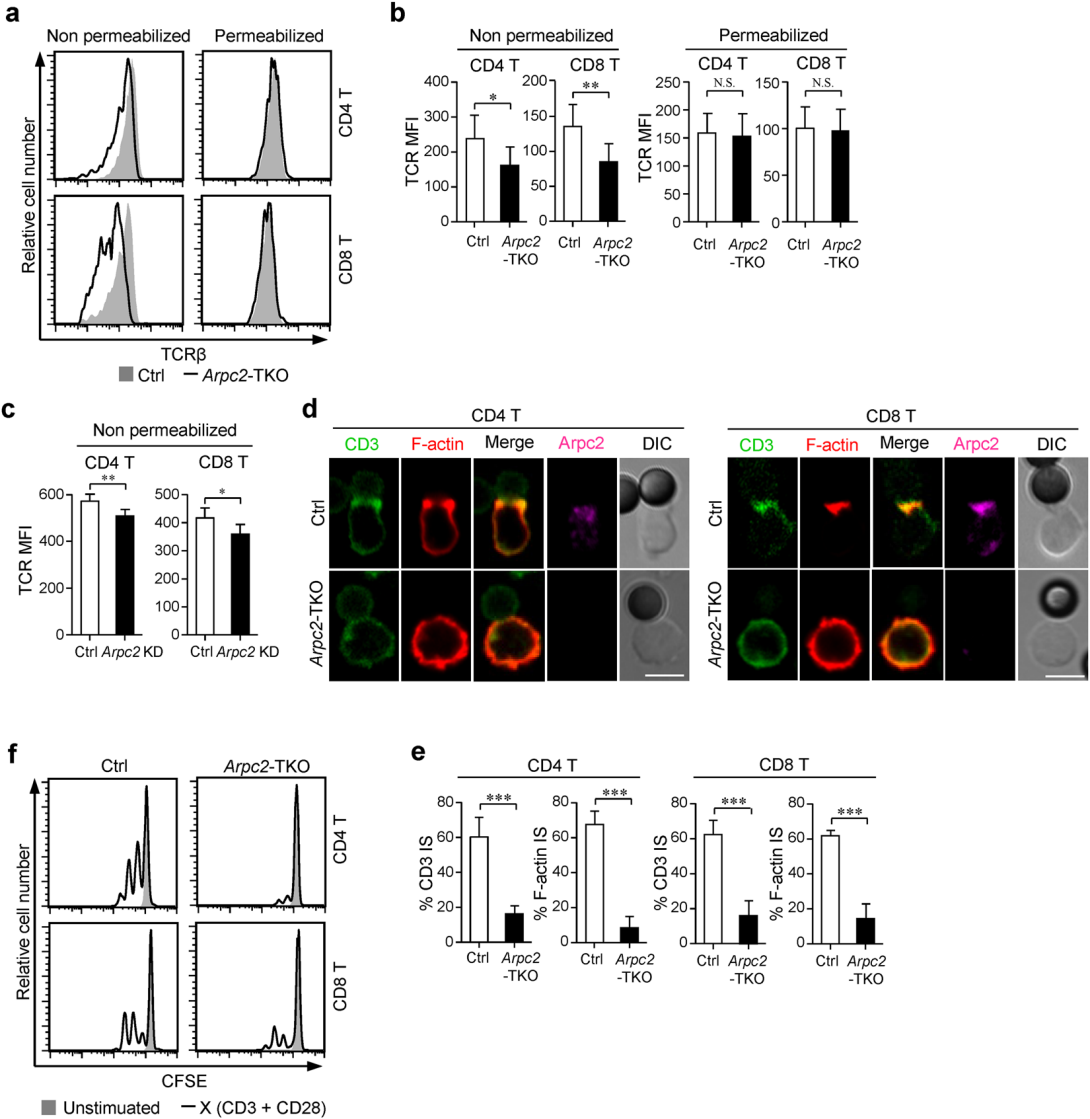
Peripheral *Arpc2-*TKO T cells show impaired surface TCR maintenance and immune synapse formation. (**a**) Representative histograms and (**b**) Mean fluorescence intensity (MFI) of surface (n = 7) and total (n = 5) TCRβ levels in peripheral T cells obtained from Ctrl and *Arpc2*-TKO mice. (**c**) MFI of surface TCRβ levels of control and Arpc2 knockdown peripheral T cells (n = 5) (**d**) T cells isolated from the lymph nodes of Ctrl and *Arpc2*-TKO mice were incubated with anti-CD3 and anti-CD28 activator beads for 15 min and stained with Phalloidin-Tritc and primary antibodies against Arpc2 and CD3ζ. They were then incubated with the appropriate secondary antibodies and imaged using microscopy. Bar is 5 μm. Representative images are shown. (**e**) The histogram show the percentage of conjugates harboring CD3ζ and F-actin at the immune synapse. At least 30 cells were analyzed for each experiment. (experiment, n = 5). (**f**) Lymphocytic T cells were labeled with CFSE and they were unstimulated or stimulated with anti-CD3 and anti-CD28 activator beads at a 2:1 (cell: bead) ratio for 48 h. The data are means ± S.D., for all panels: *P < 0.05; **P < 0.01; ***P < 0.001 by Student’s *t*-test, N.S.: no significance. All results are representative from at least three independent experiments.

Because TCR-proximal signals were reduced in the absence of Arpc2, we next analyzed TCR expression in *Arpc2*-TKO T cells and cells obtained from littermate controls. Intriguingly, FACS analysis revealed that surface TCR levels were lower in *Arpc2*-TKO T cells than in Ctrl mice, while total TCR levels were equal (Fig. 4a,b). To determine whether Arpc2 was able to directly affects surface TCR levels in peripheral T cells, we knocked down Arpc2 in peripheral T cells obtained from Ctrl mice and observed that the expression of surface TCR was also lower in Arpc2 knocked down T cells compared with control (Fig. 4c and Supplementary Fig. S4). Additionally, we found that *Arpc2*-TKO T cells failed to form immune synapse when stimulated with anti-CD3 and anti-CD28 activator beads (Fig. 4d,e). Consistent with this result, *Arpc2*-TKO T cells proliferated more slowly on TCR stimulation (Fig. 4f).

### Arp2/3 complex-promoted branched actin polymerization is required for surface TCR maintenance via modulating the trafficking of TCR^+^ endosomes

**Figure 5:**
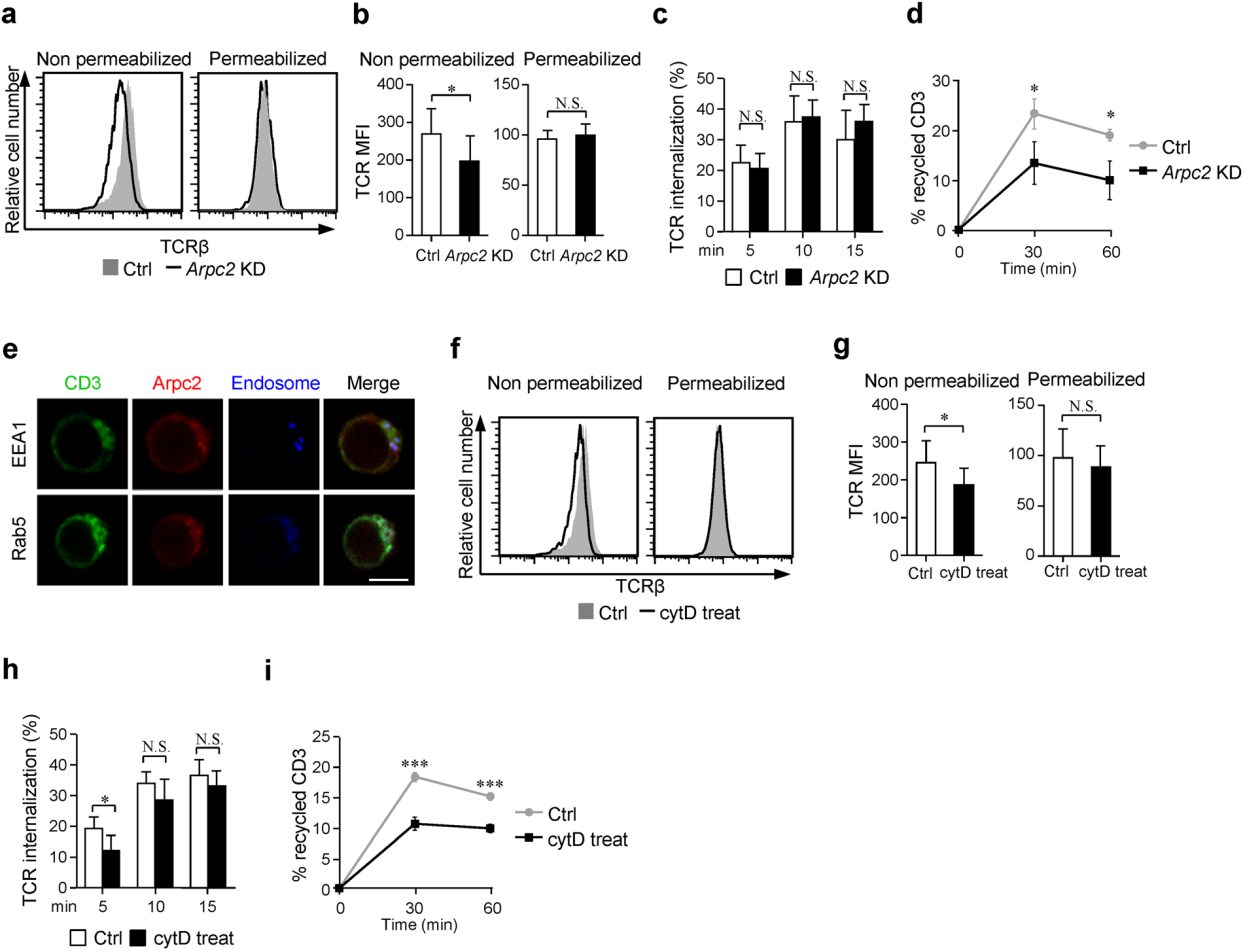
Arp2/3 complex promoted branched actin polymerization is required for surface TCR maintainance via regulating TCR^+^ endosome trafficking. (**a**) Flow cytometry analysis of surface and total TCRβ levels. (**b**) Histogram showing the MFI of surface (n = 13) and total TCRβ (n = 10) levels. (**c**) Control or *Arpc2* KD Jurkat T cells were stained with anti-TCRβ-647 on ice after they were crosslinked at 37 °C for the indicated times. The cells were then stripped and analyzed for TCR internalization using FACS (n = 5). (**d**) Flow cytometry analysis of internalized TCR recycling in control or *Arpc2* KD Jurkat T cells. The data are presented the percentage of internalized TCR receptors that had recycled back to the cell surface. (n = 3). (**e**) Immunofluorescence analysis of the location of CD3e (greeen), Arpc2 (red) and EEA1/Rab5 (blue) in Arpc2-mCherry Jurkat T cells. Bar is 5 μm. At least 30 cells were analyzed and representative images are shown. (**f**) Flow cytometry and (**g**) MFI analysis of TCR surface (n = 8) and total (n = 8) level in cytD treated Jurkat T cells compared with control cells. (**h**) Control or cytD treated Jurkat T cells were stained with anti-TCRβ-647 on ice, after crosslinking at 37 °C for the indicated times, cells were stripping and analyzed for TCR internalization by FACS. (n = 5). (**i**) Flow cytometry analysis of internalized TCR recycling in control or cytD treated Jurkat T cells. The data are presented as the percentage of internalized TCR receptors that have recycled back to the cell surface. (n = 3). The data are means ± S.D., for all panels: *P < 0.05; ***P < 0.001 by Student’s *t*-test, N.S.: no significance. All results are representative from at least three independent experiments.

Given that Jurkat T cells are bigger in size and have larger cytoplasm than primary T cells. To further assess the specific role of Arpc2 in T cells, we generated shRNA to stably silence *Arpc2* in Jurkat T cells. Non-specific shRNA-transfected cells were used as a control. Arpc2 was efficiently deleted according to an analysis of mRNA and protein levels (Supplementary Fig. S5a,b). Consistent with the aforementioned data, we also detected lower surface TCR levels (Fig. 5a,b), implying that the TCR/CD3 complex were not efficiently trafficked to the plasma membrane and therefore resided in the cytoplasm^36^. Surface TCR levels are regulated by TCR internalization and recycling from the intracellular endosomal pool^10^. We performed TCR internalization assay^18^ and found that TCR internalization was normal in *Arpc2* KD Jurkat T cells (Fig. 5c). Next, we performed TCR receptor recycling assay by using an antibody-based assay to track the recycled TCRs that had been internalized from the plasma membrane following anti-CD3 mAb crosslink at 37 °C for 2 hours^20^. As expected, FACS revealed that the *Arpc2* KD Jurkat T cells showed limited TCR recycling back to the plasma membrane (Fig. 5d), indicating that TCR recycling was impaired in the absence of Arpc2. The sustained delivery of TCR^+^ endosomes has been shown to play a central role in maintaining constant surface TCR levels in T cells^10, 16, 37^. Intriguingly, Arpc2 was spatially associated with the cytoplasmic TCR/CD3 complex, which resides in endosomes that can be labeled by EEA1 and Rab5 in Jurkat T cells using immunofluorescence assays (Fig. 5e). Thus, we presumed that Arp2/3 complex controls surface TCR maintenance in T cells by modulating the trafficking of TCR^+^ endosomes.

In agreement with previous studies that have shown that the Arp2/3 complex can nucleate branched actin filaments and promote actin cytoskeleton rearrangement, we found that *Arpc2* KD Jurkat T cells produced fewer extended actin-rich lamellipodia in a TCR-stimulated spreading assay^38^ (Supplementary Fig. S6a). We also visualized the architecture of the actin filaments network in unroofed *Arpc2* KD Jurkat T cells using scanning electron microscopy (SEM). *Arpc2* KD Jurkat T cells were much more sparsely coated with F-actin than were the controls after activation by anti-CD3 mAb (Supplementary Fig. S6b). Combined of these findings, we hypothesized that actin filaments nucleated by Arp2/3 complex might modulate the trafficking of TCR^+^ endosomes in T cells. To further evaluate whether Arp2/3 complex promoted actin filaments regulates TCR^+^ endosomes transport to the plasma membrane, we used 10 μM actin-depolymerization agent Cytochalasin D (cytD), which predominantly binds to actin filament barbed ends and compromises branched actin filaments generating^32^, to treat Jurkat T cells for 1 h. Similar to the previously described results, surface TCR levels were decreased following cytD treatment, but total TCR levels were equal (Fig. 5f,g). In accordance with aforementioned results, TCR internalization was only slightly lower (Fig. 5h) and only limited recycling of the TCRs that were internalized from the plasma membrane was observed after treatment with cytD (Fig. 5i). Altogether, these results indicate that Arp2/3 complex-nucleated actin filaments control TCR^+^ endosome trafficking to the plasma membrane to maintain constant surface TCR levels in resting state in T cells.

### Arp2/3 complex-promoted branched actin polymerization modulates the polarization of TCR^+^ endosomes during immune synapse formation

**Figure 6:**
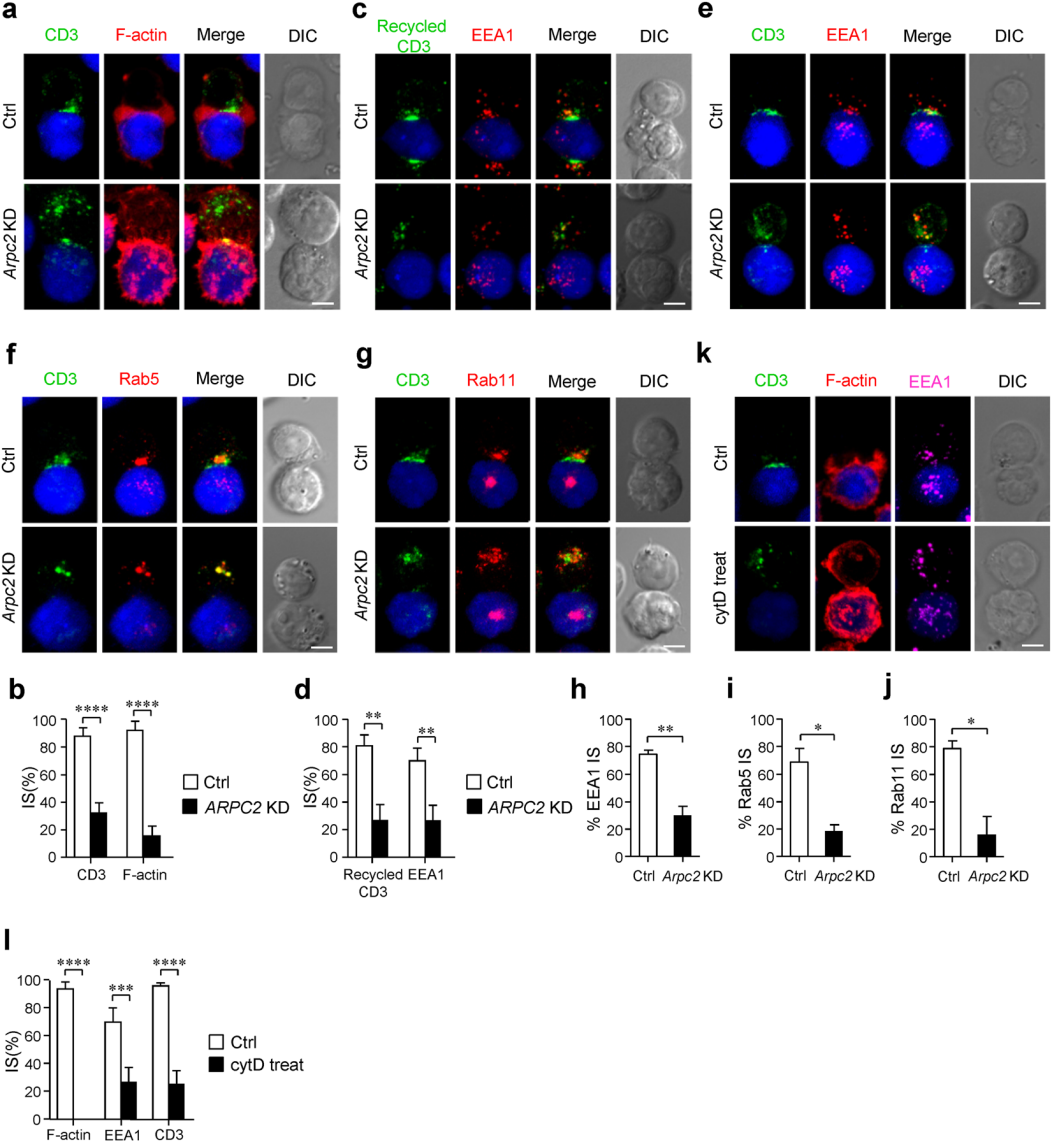
Arp2/3 complex-promoted branched actin polymerization regulates the polarization of TCR^+^ endosomes during immune synapse formation. (**a**) Immunofluorescence analysis of CD3ζ (green) and F-actin (red) localization in conjugates of Control or *Arpc2* KD Jurkat T cells with SEE-pulsed Raji cells (blue). Bar is 5 μm. (**b**) The histogram shows the percentage of conjugates that harbored CD3ζ and F-actin at the immune synapse. At least 30 cells were analyzed for each experiment. (experiment, n = 4). (**c**) Immunofluorescence analysis of EEA1 (red) and internalized CD3 (green) moved to immune synapse in conjugates pulsed with SEE-loaded Raji B cells (blue). Bar is 5 μm. (**d**). The histogram shows the percentage of conjugates that harbored EEA1 and recycled CD3 at the immune synapse. At least 30 cells were analyzed for each experiment. (experiment, n = 3) (**e–g**) Immunofluorescence analysis of (**e**) EEA1/ (f) Rab5/ (**g**) Rab11 (red) and CD3ζ (green) in Control and *Arpc2* KD Jurkat T cells in conjugates with SEE-pulsed Raji B cells (blue). Bar is 5 μm. (**h–j**) The histogram shows the percentage of conjugates harboring (**h**) EEA1/ (**i**) Rab5/ (**j**) Rab11 at the immune synapse. At least 30 cells were analyzed for each experiment. (experiment, n = 3) (**k**) Immunofluorescence analysis of CD3ζ (green) and F-actin (red) in conjugates of control or cytD treated Jurkat T cells with SEE-pulsed Raji B cells (blue). Bar is 5 μm. (**l**) The histogram shows the percentage of conjugates that harbored CD3ζ and F-actin at the immune synapse At least 30 cells were analyzed for each experiment. (experiment, n = 4). The data are means ± S.D., for all panels: *P < 0.05; **P < 0.01; ***P < 0.001; ****P < 0.0001 by Student’s *t*-test. All results are representative from at least three independent experiments.

Consistent with the aforementioned data in primary T cells, we also observed impaired immune synapse formation in Arpc2-deficient Jurkat T cells by using superantigen pulsed Raji B cells as APCs (Fig. 6a,b). We tracked the fate of internalized TCRs during immune synapse formation^19^. As expected, the internalized TCR^+^ endosomes were polarized at the T-APC contact region in control cells, whereas *Arpc2* KD Jurkat T cells exhibited a disordered endosomal pattern (Fig. 6c,d). Moreover, we found that TCR resides in endosomes labeled with EEA1 or Rab5 or Rab11 failed to polarize towards T-APC contact region in *Arpc2* KD Jurkat T cells compared with control cells (Fig. 6e-j). To further confirm that actin filaments directed TCR^+^ endosomal transport during immune synapse formation, we treated Jurkat T cells with cytD and found that the formation of immune synapse was also impaired and that TCR^+^ endosomes did not polarize to T-APC contact region (Fig. 6k,l). Together, these data indicate that Arp2/3 complex-promoted actin filaments are required for the polarization of TCR^+^ endosomes during immune synapse formation.

### Reduced surface TCR expression but not impaired immune synapse formation accounts for compromised peripheral T cell homeostasis in *Arpc2*-TKO mice

**Figure 7:**
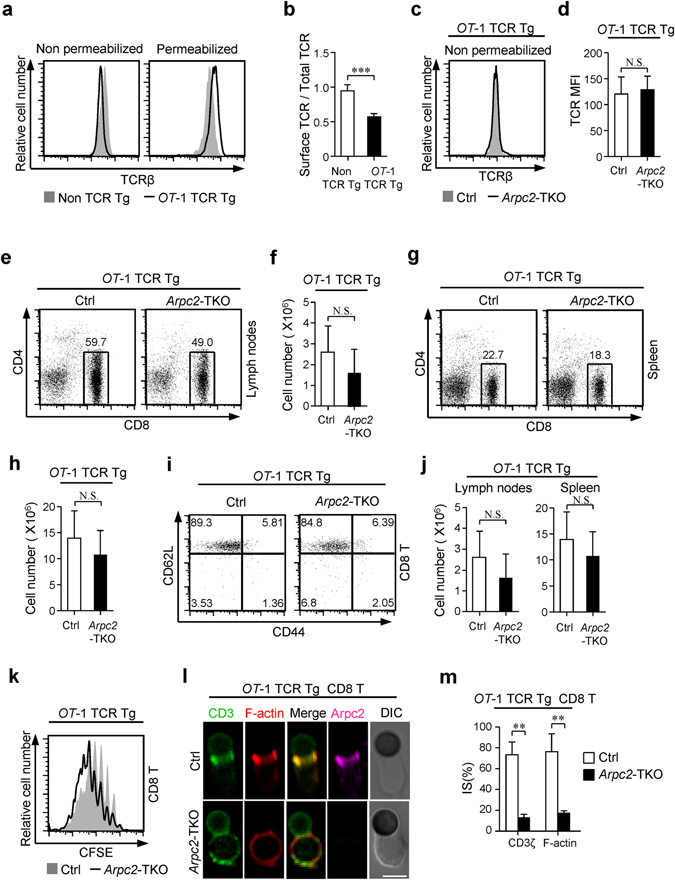
Reduced surface TCR levels but not impaired immune synapse formation accounts for compromised peripheral T cell homeostasis in *Arpc2-*TKO mice. (**a**) Flow cytometry analysis of surface and total TCR levels in CD8^+^ T cells of *OT*-1 TCR Tg mice and non TCR Tg mice. (**b**) The ratio of surface to total TCR levels from *OT*-1 TCR Tg mice and non TCR Tg mice. (n = 4). (**c**) Flow cytometry analysis of surface TCR levels of CD8^+^ T cells in lymph node from Ctrl *OT*-1 TCR Tg and *Arpc2*-TKO *OT*-1 TCR Tg mice. (**d**) Histogram shows the MFI of surface TCRβ level of CD8^+^ T cells in lymph node from Ctrl *OT*-1 TCR Tg and *Arpc2*-TKO *OT*-1 TCR Tg mice. (n = 6). (**e–h**) Flow cytometry analysis in CD4 and CD8 expression of T cells in (**e**) lymph nodes, (**g**) spleen obtained from Ctrl *OT*-1 TCR Tg and *Arpc2*-TKO *OT*-1 TCR Tg mice. Total numbers of CD8^+^ T cells in (**f**) lymph nodes (n = 9) and (**h**) spleen (n = 7). (**i**) Expression of CD44 and CD62L on T cells from Ctrl *OT*-1 Tg and *Arpc2*-TKO *OT-*1 Tg mice. (**J**) Total number of CD8^+^ naive T cell in lymph nodes (n = 8) and spleen (n = 6) were quantified from Ctrl *OT*-1 Tg and *Arpc2*-TKO *OT-*1 Tg mice. (**k**) Naïve CD8^+^ T cells isolated from Ctrl or *Arpc2*-TKO *OT*-1 TCR Tg mice were labeled with CFSE and transferred into *Rag1* ^−/−^ mice. After 7 days, the mice were sacrificed, and the lymphocytes were analyzed using FACS. (**l**) Isolated CD8^+^ T cells from the lymph nodes of Ctrl and *Arpc2*-TKO *OT-*1 TCR Tg mice were incubated with anti-CD3 and anti-CD28 activator beads for 15 min and then co-stained antibodies against CD3ζ, phalloidin and Arpc2. They were then imaged using microscopy. Bar is 5 μm. Representative images are shown. (**m**) Histogram shows the percentage of conjugates that harbored CD3ζ and F-actin at the immune synapse. At least 30 cells were analyzed for each experiment. (experiment, n = 3). The data are means ± S.D., for all panels: **P < 0.01; ***P < 0.001 by Student’s *t*-test, N.S.: no significance. All results are representative from at least three independent experiments.

Our aforementioned data demonstrate that *Arpc2*-TKO T cells exhibit lower surface TCR levels. Peripheral T cells obtained from *OT*-1 TCR transgenic (Tg) mice have a lower ratio of surface to total TCR than non TCR transgenic Ctrl mice (Fig. 7a,b), which indicated that surface TCR levels are much easier to get saturation in *OT*-1 TCR Tg T cells than non TCR Tg T cells. Thus, lower surface TCR levels requirements and higher total TCR expression in *OT*-1 TCR Tg T cells might weaken Arp2/3 complex mediated TCR translocation effect in maintaining surface TCR levels. So we generated *Arpc2*-TKO *OT*-1 TCR Tg mice and observed that surface TCR levels were restored in *Arpc2*-TKO *OT*-1 TCR Tg mice compared with Ctrl *OT*-1 TCR Tg mice (Fig. 7c,d). Intriguingly, the peripheral T cell populations could be restored to a great extent in both lymph nodes and spleen (Fig. 7e-h) and that the naïve T cells population was also restored (Fig. 7i,j). Moreover, the T cell homeostatic proliferation potential in *Arpc2*-TKO *OT*-1 TCR Tg mice was equal compared with Ctrl *OT*-1 TCR Tg mice by adoptive transfer assay (Fig. 7k). The signaling, proliferation and apoptosis was also similar (Supplementary Fig. S7a-e). However, peripheral T cells obtained from *Arpc2*-TKO *OT*-1 TCR Tg mice were still unable to form immune synapse (Fig. 7l,m). These results indicate that immune synapse is not necessary for T cell homeostasis. Collectively, these results lead us to conclude that the defective homeostasis observed in *Arpc2*-TKO T cells is mainly caused by reduced surface TCR levels and not impaired immune synapse formation.

## Discussion

T cell homeostasis is essential for the functions of the adaptive immune system because it maintains a constant pool of peripheral T cells. This process is regulated by homeostatic signals that are initiated following the recognition of the self-peptide-MHC complex and IL-7 signals^1^. Although TCR is important for T cell homeostasis and functions, how surface TCR levels is regulated and its biological significance on T cells remain largely unknown. In this study we explored the role of the actin-polymerized complex Arp2/3 in TCR trafficking and peripheral T cell homeostasis. We have demonstrated that Arp2/3 complex-nucleated actin filaments promote TCR^+^ endosomes movement to sustain homeostatic signaling by maintaining the constant cell surface level of TCR. Notably, such TCR levels but not immune synapse are essential for T cell homeostasis. Thus our study provides unique insights into the relationship among actin filaments-promoted TCR^+^ endosome trafficking, surface TCR maintenance and T cell homeostasis.

In recent years, TCR recycling has been shown to play a central role in maintaining surface TCR levels which mainly depends on the continuous delivery of the TCR/CD3 complex from an intracellular endosomal pool^16^. In our study we have demonstrated that Arp2/3 complex-promoted actin polymerization is essential to this process. As we have shown, TCRs are continuously internalized and recycled back to the plasma membrane^10^, thus proteins involved in vesicle docking and fusion may concentrate at particular areas underneath plasma membrane, where they form an “active zones”. Since actin filaments are located in “active zones”, they may also help to restrict and stabilize the proteins that are associated with endosomal transport in this area so as to facilitate the recycling TCR^+^ endosomes to plasma membrane. Matteoni *et al*. found that endosomes moved along microtubules and that this process was compromised by treatment with nocodazole^39^. Gautreau *et al*. showed that WASH co-localized with endosomes in the NIH3T3 cell line^40^. These findings also support the notion that the cytoskeleton plays a role in promoting endosomal TCR trafficking and that these biological processes could be important to T cells. Here we found that *Arpc2* is spatially associated with TCR^+^ endosomes and that Arp2/3 complex-promoted actin polymerization is required for TCR^+^ endosomes trafficking to maintain the constant surface TCR levels. However, we have not determined whether TCR^+^ endosome movement also depends on crosstalk between microtubules and actin filaments.

An accumulating amount of evidence indicates that the recycled endosomal receptor pool is the source of the constitutive transfer of the TCR-CD3 complex to the plasma membrane. This pool enables T cells to rapidly upregulate surface TCR levels and to sustain TCR signals when they come into contact with antigens^16^. A number of other TCR signaling molecules, including LAT and Lck, also sustain TCR signaling as two pools: one that is plasma membrane associated and another that exists as an intracellular endosomal pool^41, 42^. During antigen-induced immune synapse formation, microtubule-organizing center (MTOC) forms towards T-APC contact region and microtubules radiating from it set the track for directional transport of recycling TCRs^43^. Actin filaments-promoted TCR^+^ endosomes trafficking is also important during the process of antigen induced T cell activation. Numerous TCR signaling molecules, such as VAV1, CDC42 and NPFs, drive the Arp2/3 complex mediated actin filaments nucleation after T cell activation^30^. In this report, we have also demonstrated that TCRs resided in endosomes marked by EEA1, rab5 and rab11 are polarized towards immune synapse under the guidance of Arp2/3 complex-nucleated actin filaments. Thus, we presume that Arp2/3 complex-nucleated actin filaments direct TCR^+^ endosomes polarization to the immune synapse and maintain surface TCR levels, which further sustain TCR signals and strengthen immune synapse.

It has been established that naive T cell homeostasis relies on signals from self-peptide-MHC complexes. Previous study has found that the intense scanning of DCs by T cells in the absence of foreign is essential for T cell survival and function in steady state. Low-level TCR contact with self-antigens can promote T cell survival but is insufficient to induce their proliferation^34^. However, the strength of TCR signals that is required for T cell homeostasis is largely unknown. Here we demonstrate that Arp2/3 complex nucleated-actin filaments could direct TCR^+^ endosome trafficking and thus maintain constant surface TCR levels, which is vital to T cell homeostatic signals. In this study, we found that the absolute number of CD8^+^ T cells in *Arpc2*-TKO *OT*-1 TCR Tg mice was partially rescued to levels similar to those in Ctrl *OT*-1 TCR Tg mice, while immune synapse formation remained impaired. These results indicate that homeostatic signals generated from the Arp2/3 complex promote the constant supply of surface TCR, but signals from immune synapses are not vital for T cell homeostasis.

We have also demonstrated that the role of the Arp2/3 complex in T cells is developmental independent in thymus. Peripheral T cells exhibit higher transcription level of Arpc2 than thymocytes, and Arp2/3 complex-promoted actin nucleation is essential for peripheral T cell homeostasis but does not affect T cell development in the thymus. In addition, peripheral T cells have a much longer life span about two months^44, 45^, and they are harder to get close to scanning Dendritic cells (DC) pulsed with self-peptide. Whereas, thymocytes divide through developmental stages and they are near to self-peptide presented thymic stromal cells^46^. We speculate that peripheral T cells may need such post-translational regulation of surface TCR levels more than thymocytes, that’s why actin filaments regulated TCR^+^ endosomes trafficking might not influence thymocytes development in thymus, but of great importance in peripheral T cells.

Hence, our data suggest a mechanism by which the actin filaments regulate T cell homeostasis via regulating TCR^+^ endosomes recycling to maintain surface TCR levels. Given the importance of the actin filaments to T cell functions, our data explore a new concept in which the constant TCR signal transduces from a self-peptide-MHC complex instead of forming a functional immune synapse is required for T cell homeostasis.

## Methods

### Mice

The C57BL/6 sperm with conditional deletion of *Arpc2* alleles were ordered from the European Conditional Mouse Mutagenesis Program consortium as described^32^. *Arpc*2^fl/fl^ mice mated with FLP recombinase mice and then with *Cd*4*-Cre* transgenic mice. *Cd*4*-Cre* transgenic mice, *OT-*1 TCR transgenic mice, recombination-activating gene 1-deficient mice, and CD45.1 mice were obtained from The Jackson Laboratory. All mice were genotyped by PCR. Mice aged 5–8 weeks were used for experiments. All animal experiments were conducted in compliance with National Institutes of Health guidelines and were approved by the institutional animal care and use committee of the Shanghai Institutes for Biological Sciences (Chinese Academy of Sciences).

### Antibody and reagents

For the flow cytometry analysis, anti-CD4 (GK1.5), anti-CD8α (53–6.7), anti-CD44 (IM7), anti-CD127 (A7R34), anti-CD69 (FN50), anti-Bcl-2 (BCL/10C4) were purchased from Biolegend. Anti-CD62L (MEL-14), anti-CD69 (H1.2F3), anti-CD5 (53–7.3), anti-CD45.1 (A20), anti-CD45.2 (104), mouse lgG1 κ isotype control (556027), anti-CD25 (PC61), Rat lgG2a isotype control (eBRG1) and purified anti-human-CD3 (OKT3) were purchased from BD Bioscience. Anti-Foxp3 (FJK-16s), anti-TCRβ (H57–597) and anti-CD3 (UCHT1) were purchased from eBioscience. Fluo-4,AM (F14217) was purchased from Invitrogen. Streptavidin (NEBN7021S) was purchased from Sigma.

For western analysis, anti-α-tubulin (sc-53029) and anti-Arp3 (sc15390) antibodies were purchased from Santa Cruz Biotechnology. Anti-Arpc2 (ab96779) was purchased from Abcam.

For immunofluorescence analysis, anti-Mouse TCR β-chain-488 (H57-597) and anti-Mouse CD3-488 (17A2) were purchased from Biolegend. Anti-CD3ζ (6B10.2) was purchased from Santa Cruz. Anti-EEA1 (C45B10), anti-Rab5 (C8B1) and anti-Rab11 (D4F5) were purchased from Cell Signaling Technology. Anti-LAMP1 (ab24170) was purchased from Abcam.

Cytochalasin D (ab143484) was purchased from Abcam. Staphylococcal enterotoxin E (ET404) was purchased from Toxin Technology. CELLTRACKER(TM) BLUE CMAC (C2110) was purchased from Invitrogen.

### Cell lines and Jurkat T cells construction

For construction of knockdown Jurkat T cells, 293FT cells were co-transfected with indicated plasmids including: pLKO.1 shRNA, psPAX2 and pMD2.G. The lentiviral supernatants were harvested from cultured media 48 h after transfection. T cells maintained in RPMI-1640 were incubated in the lentiviral supernatants. Human *Arpc2*-target shRNA 5′-ACAUGGUGCUGAUGAGUUAUU-3′; scramble shRNAs AACAAGAUGAAGAGCACCAA; For construction of β-actin-mCherry or Arpc2-mCherry Jurkat T cells, 293FT cells were co-transfected with indicated plasmids including: β-actin-pHAGE-mCherry or Arpc2-pHAGE-mCherry, psPAX2 and pMD2.G.

### Knockdown primary T cells construction

For construction of knockdown peripheral T cells, Plat-E cells were transfected with MLP shRNA plasmids, retrovirus supernatants were harvested 48 h after transfection. T cells were then incubated in media containing retrovirus supernatants and GFP positive cells were used for the indicated experiments after 72 h. Mouse Arpc2-target shRNA:5′-CAGTATTTCTTTGAAATTCTA-3′; A GFP positive cells-negative control was described^47^.

### Real-time RT-PCR

Total RNA was extracted and reverse-transcribed as done previously. The mRNA levels of the indicated genes were normalized to GAPDH by using real-time RT-PCR (Rotor gene 6000; Corbett Life Sciences) with a SYBR Green QPCR Master Mix (Toyobo). Primers were used for qPCR: Arpc2-human, 5′-AGACACAGACGCCGCTGTGGGT-3′ and 5′-GTGGTAGTGCAGATAGTCCCGG-3′; Arpc2-murine, 5′-AGACACAGATGCTGCTGTGGGT-3′ and 5′-GTGGTAGTGCAGATAGTCCCGG-3′; GAPDH-human, 5′-CCAGGTGGTCTCCTCTGACTTC-3′, 5′-GTGGTCGTTGAGGGCAATG-3′; GAPDH-murine, 5′-ACTCCACTCACGGCAAATTCA-3′, 5′-GCCTCACCCCATTTGATGTT-3′.

### Electron microscopy

Control and *Arpc2* KD Jurket T cells were added to 20 μg/ml OKT3 coated glass coverlip for 15 min, then the Jurket T cells were went through fixation, dehydration, embedding, critical point drying and conductive coating and viewed under FEI Quanta 250 scanning electron microscopy.

### T-cell stimulation

For immune synapse formation, CMAC labeled SEE-pulsed Raji cells were incubated with Jurkat T cells for 5min^48^. For confocal, T cells were sorted and incubated with anti-CD3 and anti-CD28 activator beads for 15min^49^. For calcium flux assay, Naïve T cells were preloaded with Fluo4-AM and stained with anti-CD4, biotinylated anti-CD3 (10 μg/ml) and anti CD28 (10 μg/ml). Cells were washed and pre-warmed to 37 °C. After addition of streptavidin (20 μg/ml), Ca^2+^ mobility was measured by FACSAria II^50^. For naïve T cell stimulation, T cells were stimulated with anti-CD3 and anti-CD28 activator beads.

### TCR/CD3 complex recycling and internalization

Receptor recycling was determined by FACS as previously described^20^. Briefly, Jurkat T cells were stained with anti-CD3 mAb on ice, washed and moved to 37 °C for 60 min, then acid-stripped to remove surface–bound mAb and shifted at 37 °C to allow recycling of internalized mAb-TCR/CD3 complex. The recycled Receptor-mAb complexes to plasma membrane were measured after staining with anti-mouse secondary antibody conjugated to Alexa Fluor 647. The data presented the percentage of the internalized TCR. For immunofluorescence analysis, T cells were stained with OKT3 at 37 °C for 2 h. After acid-stripping, cells were incubated with SEE-pulsed Raji cells for 15 min at 37 °C. For TCR internalization analysis^18^, cells were incubated on ice with anti-TCRβ-647 for 30 min, washed and incubated at 37 °C for indicated times. After acid-stripping, cells were analyzed by FACS.

### Immunofluorescence microscopy

Cells were allowed to adhere to poly-L-Lysine-coated coverlips. Cells were then fixed with 4% (wt/vol) paraformaldehyde and permeabilized with permeabilization buffer (0.1% (vol/vol) triton X-100) in PBS, after blocking in 1%BSA in Tris-Buffered Saline and Tween 20 (TBST), cells were then stained with indicated primary antibodies and then incubated with indicated secondary antibodies. Slides were analyzed on Leica TCS SP8 and Olympus FV1200 confocal microscopy.

### Adoptive Transfer of Naïve T Cells

The adoptive transfer of naïve T cells was done as previously described^33^. The sorting naïve CD4^+^ and CD8^+^ T cells from 6- to 8-week-old *Arpc2*-TKO mice were mixed with equal number of T cells from CD45.1^+^ C57BL/6 control mice. After labeled with CFSE, 1 × 10^6^ cells were injected into tail vein to 6- to 8-week-old *Rag1* ^−/−^ mice. Mice were sacrificed 7 days after transfer. CFSE dilution of Ctrl and *Arpc2*-TKO T cells in lymph nodes were measured by LSRII. All the FACS data were analyzed by FlowJo software.

### Statistics

All experiments described were performed three or more times. Average values were expressed as mean ± S.D. The two-tailed unpaired Student’s *t*-test was used to determine statistical significance unless otherwise indicated.

## Electronic supplementary material

Supplementary information
