# Supplementary material for: Arp2/3 complex controls T cell homeostasis by maintaining surface TCR levels via regulating TCR+ endosome trafficking
Source: Sci Rep. 2017 Aug 21;7:8952. doi: 10.1038/s41598-017-08357-4 (PMC5566485; doi:10.1038/s41598-017-08357-4)
Supplement: Supplementary file 1 — Supplementary information [file 41598_2017_8357_MOESM1_ESM.pdf]

Supplemental Information

Arp2/3 complex controls T cell homeostasis by maintaining surface TCR levels via regulating TCR$^+$ endosome trafficking

Ye Zhang[a,b1], Hao Shen[a,b1], Haifeng Liu[a,b], Haiyun Feng[a,b,c], Yan Liu[a,b,c], Xiaoyan Zhu[a,b] and Xiaolong Liu[a,b,c*]

[a]State Key Laboratory of Cell Biology, CAS Center for Excellence in Molecular Cell Science, Institute of Biochemistry and Cell Biology, Shanghai Institutes for Biological Sciences, Chinese Academy of Sciences, Shanghai 200031, China

[b]University of Chinese Academy of Sciences, 320 Yue-Yang Road, Shanghai 200031, China.

[c]School of Life Science and Technology, ShanghaiTech University, Shanghai, 200031, China

[1]These authors contributed equally to this work

[*]Correspondence should be addressed to X.L. (liux@sibs.ac.cn)

Phone: 86-21-54921176

FAX: 86-21-54921178

Supplementary Figure 1

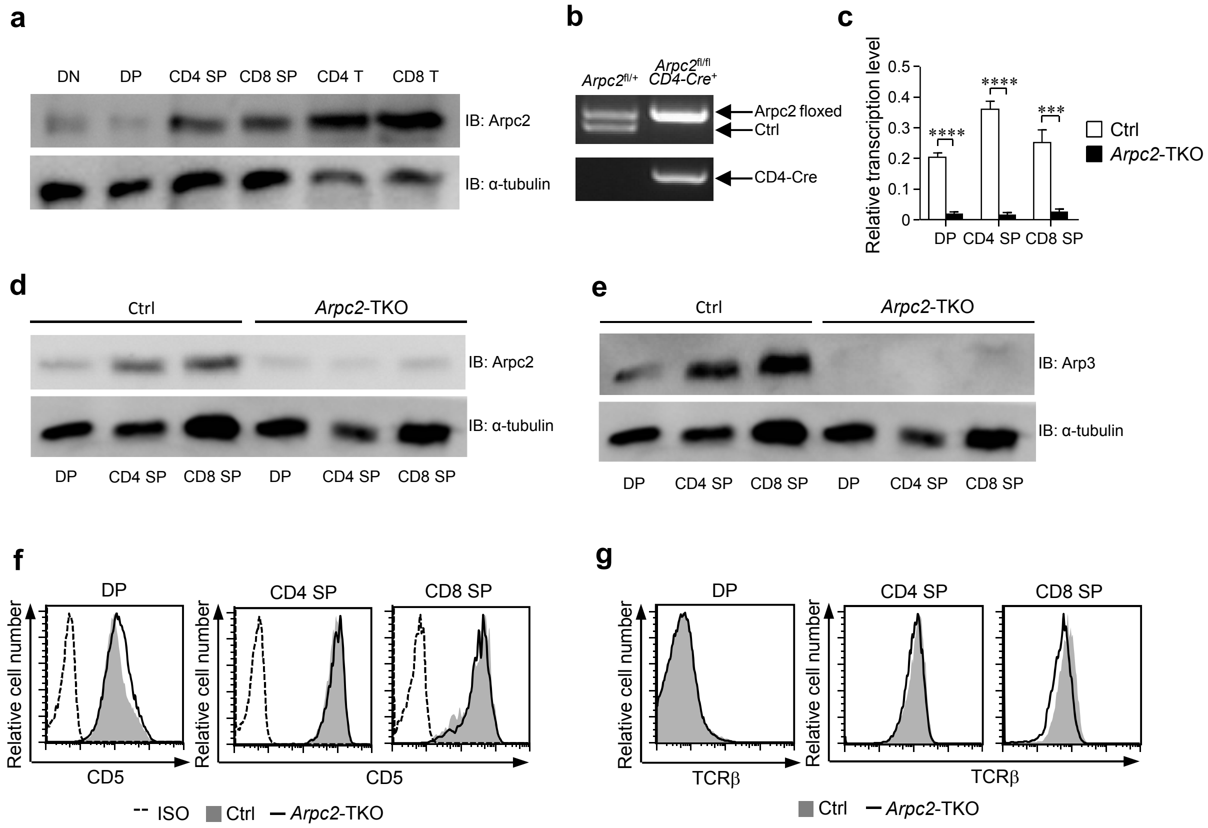

Supplementary Figure 1. Specific deletion of Arpc2 in T cells.

(**a**) Protein level of Arpc2 in DN, DP, CD4 SP, CD8 SP, CD4 T and CD8 T cells in control mice. The expression of total a-tubulin served as a loading control. (**b**) Genomic DNA was extracted from tails of control mice (*Arpc2*$^{fl/+}$) and Arpc2-deficient (*CD4-cre*$^{+}$*Arpc2*$^{fl/fl}$) mice and then were analyzed by PCR. (**c**) The relative expression level of Arpc2 in DP, CD4 SP and CD8 SP thymocytes obtained from Ctrl or *Arpc2*-TKO mice. (n=3; ***P < 0.001 and ****P < 0.001 by two-tailed unpaired *t*-test). (**d**) Analysis of Arpc2 protein levels in DP, CD4 SP and CD8 SP thymocytes. The expression of total a-tubulin served as a loading control. (**e**) Analysis of Arp3 protein levels in DP, CD4 SP and CD8 SP thymocytes. The expression of total a-tubulin served as a loading control. (**f**) Flow cytometry analysis of the surface markers CD5 in DP and SP thymocytes obtained from Ctrl and *Arpc2*-TKO mice, IgG2a isotype control were used. (**g**) Flow cytometry analysis of the surface markers TCRβ in DP and SP thymocytes obtained from Ctrl and *Arpc2*-TKO mice.

Supplementary Figure 2

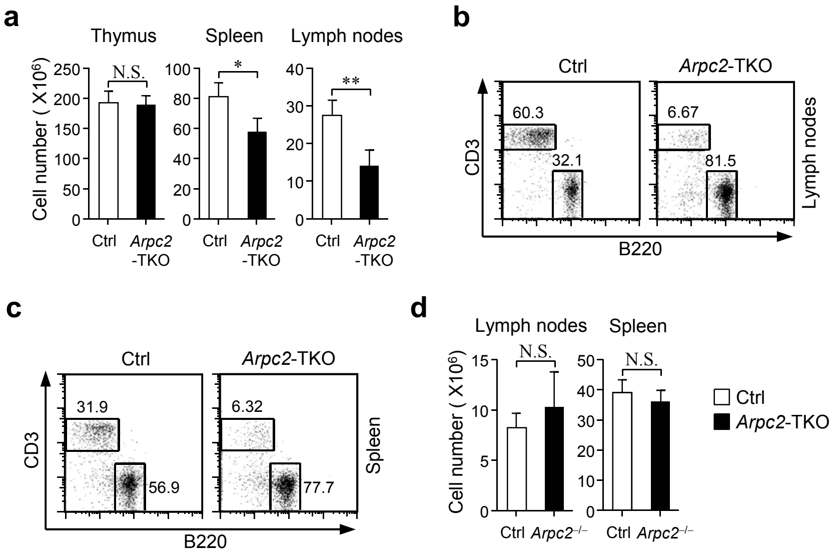

Supplementary Figure 2. Cellularity of thymus, spleen and lymph nodes.

(**a**) Cellularity of thymus, spleen and lymph nodes (n=4; *P < 0.05 and **P < 0.01 by two-tailed unpaired *t*-test; N.S.: no significance). (**b-c**) Flow cytometry analysis of CD3 and B220 expression in (**b**) lymph nodes and (**c**) spleen. (**d**) Total number of $B220^+$ B cells in lymph nodes and spleen (n=4; two-tailed unpaired *t*-test; N.S.: no significance).

Supplementary Figure 3

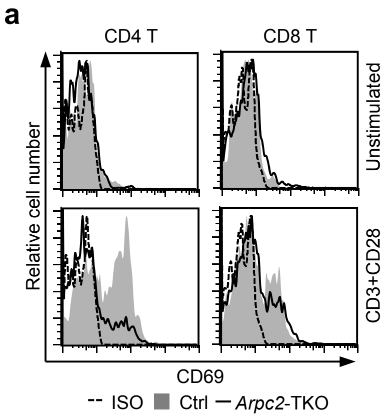

Supplementary Figure 3. Decreased T cell activation in *Arpc2*-TKO mice T cells.
(**a**) Histogram show Flow cytometry analysis of CD69 expression in T cells that were unstimulated or stimulated with anti-CD3 and anti-CD28 activator beads for 4h. IgG1, κ isotype control were used.

Supplementary Figure 4

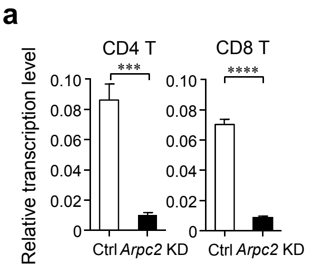

Supplementary Figure 4. Knockdown of Arpc2 in primary T cells.
(**a**) Analysis of Arpc2 mRNA level in control and Arpc2 knockdown peripheral T cells (n=3; ***$P < 0.001$ by two-tailed unpaired *t*-test). Representative images are shown. Error bars indicate s.d.. All results are representative from at least three independent experiments.

Supplementary Figure 5

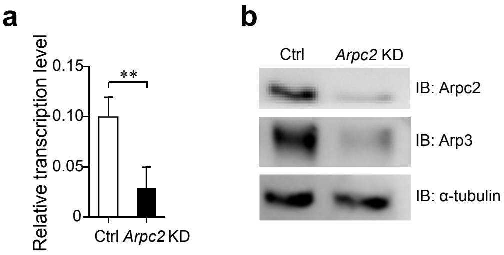

Supplementary Figure 5. Arpc2 was efficiently deleted in Jurkat T cells.
(**a**) Analysis of Arpc2 mRNA (n=4; **P < 0.01 by two-tailed unpaired *t*-test) and (**b**) protein levels in Control and *Arpc2* KD Jurkat T cells. Representative images are shown. Error bars indicate s.d.. All results are representative from at least three independent experiments.

Supplementary Figure 6

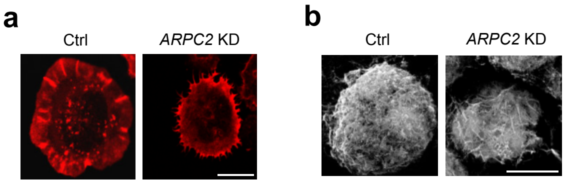

Supplementary Figure 6. Loss of Arpc2 leads to defective branched actin polymerization in T cells.

(**a**) β-actin-mCherry Jurkat T cells (red) were transfected with control or sh*Arpc2* and plated on coverslips coated with anti-CD3mAb for 15 min. They were then imaged using microscopy. Bar is 10 µm. At least 30 cells are imaged. Representative images are shown. (**b**) Control and Arpc2 KD Jurkat T cells were allowed to spread for 15 min at 20 µg/ml OKT3 on coated coverslips. The cells were then unroofed and photographed to observe the organization of F-actin under SEM. Bar is 4 µm. At least 30 cells were imaged. Representative images are shown. All results are representative from at least three independent experiments.

Supplementary Figure 7

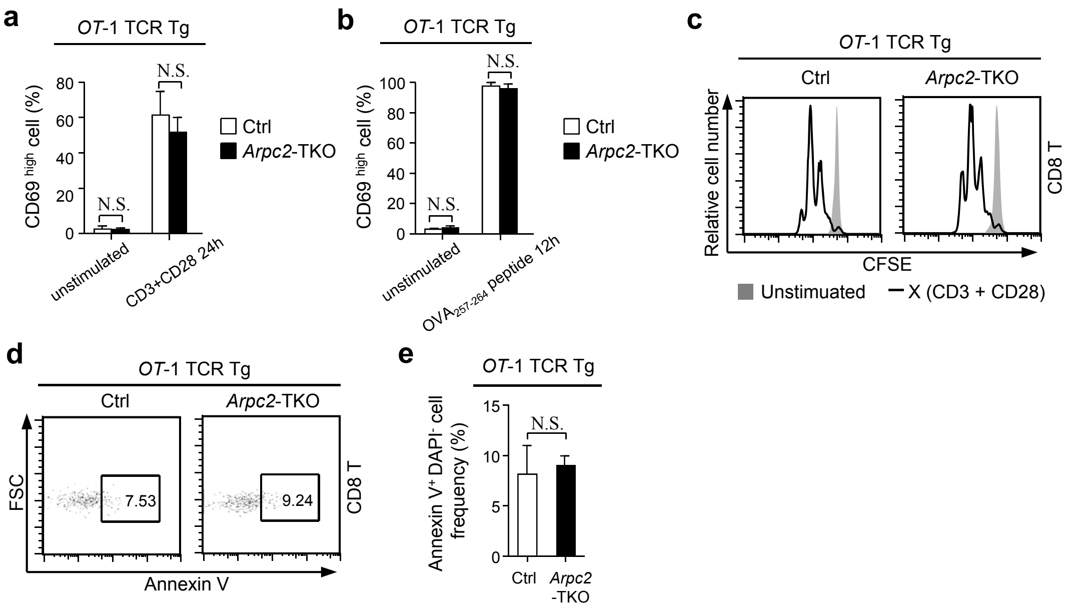

Supplementary Figure 7. The signaling, proliferation and apoptosis was normal in *OT*-1 TCR Tg *Arpc2*-TKO T cells.

(**a**) Flow cytometry analysis of CD69 expression in *OT*-1 TCR Tg T cells obtained from Ctrl and *Arpc2*-TKO mice that were unstimulated or stimulated with anti-CD3 and anti-CD28 activator beads for 24 h (n=3; two-tailed unpaired *t*-test; N.S.: no significance) or (**b**) with antigen presenting cells pulsed SIINFEKL ($OVA_{257\text{-}264}$) peptide for 12h (n=4; two-tailed unpaired *t*-test; N.S.: no significance). (**c**) Lymphocytic T cells of *OT*-1 TCR Tg T cells obtained from Ctrl and *Arpc2*-TKO mice were labeled with CFSE and they were unstimulated or stimulated with anti-CD3 and anti-CD28 activator beads for 48 h. (**d**) Survival was assessed of *OT*-1 TCR Tg naïve T cells using flow cytometry analysis with Annexin V and DAPI staining. (**e**) The percentage statistics for Annexin V are illustrated. (n=3; two-tailed unpaired *t*-test; N.S.: no significance).
